# Classical and New Approaches to Health Risk Assessment of Acrylamide Through the Consumption of Potato Chips

**DOI:** 10.1002/fsn3.71137

**Published:** 2025-10-30

**Authors:** Kiana Afsari, Farzad Kobarfard, Hassan Yazdanpanah, Anca Oana Docea, Christina Tsitsimpikou, Samira Eslamizad, Aristides Tsatsakis

**Affiliations:** ^1^ Department of Medicinal Chemistry, School of Pharmacy Shahid Beheshti University of Medical Sciences Tehran Iran; ^2^ Food Safety Research Center Shahid Beheshti University of Medical Sciences Tehran Iran; ^3^ Department of Toxicology and Pharmacology, School of Pharmacy Shahid Beheshti University of Medical Sciences Tehran Iran; ^4^ Department of Toxicology University of Medicine and Pharmacy of Craiova Craiova Romania; ^5^ General Chemical State Laboratory of Greece Athens Greece; ^6^ Center of Toxicology and Science Applications, Medical School University of Crete Heraklion Greece; ^7^ Universidad Ecotec Samborondon Ecuador; ^8^ Sechenov IM First State Medical University Moscow Russia

**Keywords:** carcinogenic potency, chronic oral exposure, food safety, food toxicology, health risk assessment, LC–MS/MS

## Abstract

Potato chips (chips, UK crisps) are one of the popular snacks, particularly among children and teenagers. Chips are prone to acrylamide (AA) formation, as a suspected carcinogen. In the present study, an in‐house sample preparation procedure and liquid chromatography with tandem mass spectrometry (LC–MS/MS) method were developed and validated to monitor AA in 113 potato chip samples from Iranian brands during two periods: 2016–2017 and 2020–2021. AA risk assessment was performed by applying conventional and new approaches, using the calculations of Target Hazard Quotient (THQ), source related Hazard Quotient (HQs), Incremental Lifetime Cancer Risk (ILCR), and Margin of Exposure (MOE) for various age and gender groups. The prevalence of acrylamide contamination increased from 13% (25.1 ng/g) to 31% (31.9 ng/g) between the first and second periods. The noncarcinogenic risk index (THQ) increased in 2020–2021, approximately 20% higher than in 2016–2017, with children at higher risk. MOE values indicate that the neoplastic effects of AA in chips can be threatening in both genders, under 10 years old, as the MOE is below 10,000, and could fall into the category of the highest public health concern. New risk assessment approaches revealed that HQs were approximately six times higher in adults and five times higher in children, emphasizing the need for a comprehensive risk evaluation that considers multiple sources of AA exposure.

## Graphical abstract

Developing an in‐house method for determining acrylamide in potato chips. Monitoring acrylamide in 113 potato chip samples in two periods. Assessing the health risk of acrylamide by applying conventional and new approaches. Elevated HQs are about six times greater than HQ in adults and about five times in children. Children under 10 years of age could be at elevated carcinogenic risk.

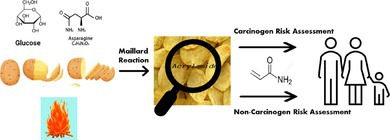

## 1 Introduction

Potato chips (chips, UK crisps), as thin and crispy potato slices, are a popular snack, particularly among children and teenagers. Approximately 750,000 tons of potatoes are produced in Iran each year, of which nearly 80% are processed into products such as potato chips, resulting in an average daily consumption of 19.3 g per person per day or 7.05 kg per person per year (Schripsema and Meijer 2017).

In April 2002, the Swedish National Food Administration highlighted significant acrylamide (AA) levels in fried or roasted starchy foods, raising public health concerns (Swedish National Food Administration 2002). AA [CH2=CH (C=O) NH2] is a toxic compound that is classified as a probable human carcinogen (Group 2A) by the International Agency for Research on Cancer (IARC) (IARC 2019), is also classified in the European Union as a carcinogen (Category 1B), mutagen (Category 1B), and reproductive toxicant (Category 2, fertility) (EC Regulation 1272/2008 Of The European Parliament And of Council 2013; European Commission 2002). In addition to the above toxic effects, one of the main toxic effects of AA on the human body is on the nervous system, and AA can also cause damage to the spleen, liver, intestine, thymus, etc. (Fan et al. 2023). AA formation in carbohydrate‐rich foods is primarily attributed to the Maillard reaction between the carbonyl group of reducing sugars and the amino acid asparagine (Rifai and Saleh 2020). The extent of AA formation can be influenced by factors such as a cooking temperature of 120°C or higher (Halford et al. 2012) and the specific type of potato with a high content of reducing sugar plays a significant role in the formation of AA (Fiselier and Grob 2005) with higher levels typically found in products that are cooked to a darker color or have been processed to enhance crispiness (Chuda et al. 2003). Among these, potato chips represent one of the highest dietary sources of AA, alongside French fries, cereal‐based products, and coffee (European Food Safety Authority (EFSA) 2015). In addition, AA is also found in other foods, including cakes, biscuits, and nuggets (a kind of carbohydrate and protein‐containing food; Aghvami et al. 2023; Hoseini Majd et al. 2025; Seilani et al. 2021). Potato chips are the largest sector in the snack food market, typically made from uniform slices of raw potatoes, 0.035–0.070 in. thick. Prefrying steps like washing or blanching remove excess starch and reduce sugars to prevent browning. Potatoes generally contain 80%–85% water and 15%–20% dry matter, primarily starch. Reducing sugar levels exceeding 2% can cause excessive browning due to the Maillard reaction, making chips visually undesirable (Pedreschi et al. 2008). In 2017, the European Commission established a benchmark level (BML) of 750 μg/kg for AA in potato chips to guide mitigation efforts (Eu Commission Regulation 2017/2158 2017). Because of the health risks associated with AA, including its potential carcinogenicity as shown in animal studies, which includes tumor formation in the brain, thyroid, testes, lung, clitoral gland, and mammary gland, as well as the genotoxicity of AA mediated by glycidamide (GA) and a potential contribution of nongenotoxic effects, its presence in food is a public health concern (Benford et al. 2022; Rice 2005).

The most widely used analytical techniques for measuring AA in food are high‐performance liquid chromatography (HPLC) and gas chromatography (GC) in conjunction with Mass Spectrometry (MS or MS/MS) due to their sensitivity and accuracy (Hu et al. 2015; Zhang et al. 2005).

Traditional risk assessment methodologies for human health and the environment are based on the risk assessment of single substances by calculating the Target Hazard Quotient (THQ), Incremental Lifetime Cancer Risk (ILCR), and Margin of Exposure (MOE) (Eslamizad, Kobarfard, Tsitsimpikou, et al. 2019; Yazdanpanah et al. 2022). However, real‐life exposure scenarios do not correspond to exposure to a single chemical, as opposed to chemical mixtures and other nonchemical stimuli. In traditional risk assessment methodologies used for the assessment of the risk posed by AA exposure, just a single chemical was considered (Eslamizad, Kobarfard, Tsitsimpikou, et al. 2019; Yazdanpanah et al. 2022). Consequently, risk characterization is required for evaluating the real‐life exposure scenario using the new methodology of source‐related Hazard Quotient (HQS) and the adversity‐specific Hazard Index (HIA) (Goumenou and Tsatsakis 2019).

This study aimed to develop and validate a rapid, accurate method for detecting AA residues in potato chips and to assess the associated health risks of AA consumption for the respective periods in the Iranian population in different demographic groups by calculating the THQ, ILCR, and MOE applied to the conventional approach, and HQs intended for a new approach for the first time. This practical approach enhances our understanding of the risks associated with AA exposure. It emphasizes the importance of methodological advancements in food safety research, particularly in assessing potential health hazards associated with dietary consumption.

## 2 Materials and Methods

### 2.1 Chemicals

AA and AA‐d3 as internal standard (ISTD) were purchased from Sigma Aldrich (St. Louis, Mo., USA). All HPLC‐grade solvents (acetonitrile and methanol) and acetic acid (96%) were purchased from Merck (Darmstadt, Germany). Potassium hexacyanoferrate and zinc sulfate were obtained from Chem Lab NV (Belgium). Primary secondary amine (PSA) and a Solid‐Phase Extraction (SPE) bulk sorbent were purchased from Agilent Technologies (USA). Ultrapure water was prepared using an Econolab water purification system (Germany).

### 2.2 Sample Collection

Between 2016 and 2017, 69 samples and 44 samples in 2020–2021 of industrially produced potato chips were gathered from supermarkets in Tehran, Iran, and kept in a freezer at −80°C. All samples were placed in a plastic bag with a net weight of 60 g and sealed with nitrogen gas to extend their shelf life. The samples were different brands in different years, with varying batch numbers or production and expiration dates spanning over a year. Before analysis, every sample was coded, homogenized in an industrial homogenizer, and kept in amber bottles at −80°C.

### 2.3 Preparation of Standards and Reagents

1 mg of AA and AA‐d3 were dissolved separately in 1 mL of distilled water to provide a stock solution (1 mg/mL) of both compounds. Intermediate standard solutions of AA‐d3 (100,000 and 5000 ng/mL) and AA (100,000 and 10,000 ng/mL) were each prepared in distilled water. 50 μL of an internal standard solution at a concentration of 250 ng/mL was added to all samples. All standard solutions were prepared in an amber volumetric flask to avoid exposure to light and stored at 4°C until use. The samples were treated as described in the sample preparation section. The Carrez I solution was prepared by dissolving 1.5 g potassium hexacyanoferrate in 10 mL of water, and the Carrez II solution by dissolving 3 g zinc sulfate in 10 mL of water.

### 2.4 Extraction and Clean up

Homogenized potato chips (1 g) were placed in a 15 mL centrifuge tube, and 2.5 mL of methanol was added. The mixture was centrifuged at 5000 rpm and 5°C for 10 min. The entire methanol extract was transferred to a 15 mL centrifuge tube, and then 100 μL of Carrez I and II solutions were added to the methanol extract. The tube was shaken with a vortex shaker for 10 s, then 100 mg of PSA was added to the tube and then shaken for 10 s. The mixture was then centrifuged for 10 min at 5000 rpm and 25°C. The entire methanol extract was transferred to an evaporator vial. The extract was evaporated under a gentle stream of nitrogen at 40°C until the volume was reduced to 100–150 μL. 500 μL of solution was made and shaken for 20 s by mixing this extract with distilled water. The extract was filtered through a 0.22 μm filter, and then 100 μL of the extract was diluted with 900 μL of distilled water in an amberlite LC–MS vial and shaken again for 20–30 s. Finally, 20 μL of the extract was injected into the LC–MS/MS system.

In this validated method, dispersive solid‐phase extraction (DSPE) is used to remove polar matrix elements, organic acids, excess water, and other components with a mixture of PSA sorbent and MgSO4. The DSPE method permits minimization of additional steps such as precipitation, centrifugation, and filtration, which decrease the manipulation and multistage preparation of the sample (Eslamizad et al. 2020, 2016).

### 2.5 Liquid Chromatography–Mass Spectrometry

Quantification of AA was performed using an LC–MS/MS system. A 1100 series HPLC system (Agilent Technologies, Courtaboeuf, France) equipped with a binary pump, an autosampler coupled to a 4500 QTRAP tandem mass spectrometer (AB Sciex, Les Ulis, France).

Analytical separation was performed on a Porasil chromatography column (300 mm × 3.9 mm i.d., 10 μm particle size) at room temperature (Thermo Fisher Scientific). Elution was performed using a mobile phase consisting of eluents A and B at a flow rate of 0.5 mL/min. Eluent A was a mixture of 0.2% (v/v) acetic acid and 98% (v/v) ultrapure water, while eluent B was acetonitrile.

Electrospray ionization was performed in positive mode (ESI+). The spray voltage was set at 5000 V. The curtain gas, spray gas, and auxiliary gas were set at 30 psi nitrogen and 35 and 45 psi air, respectively. The source gas temperature was set at 500°C. The instrument operated in multiple reaction monitoring (MRM) mode. The transition for AA was 72.0 > 55.0 (declustering potential (DP), 35 V; entrance potential (EP), 10 V; collision cell exit potential (CXP), 14 V; collision energy (CE), 15 eV) and for AA‐d3 was 75 > 58 (DP, 35 V; EP, 10 V; CXP, 14 V; CE, 15 eV). The dwell time for each transition was set to 50 ms. Data were collected and analyzed by Analyst 1.6.3 software (AB Sciex) and Microsoft Excel 2010.

### 2.6 Method Validation

The validation of the method included the following parameters: Limit of Detection (LOD), Limit of Quantification (LOQ), linearity (spike calibration method), precision (Relative Standard Deviation [RSD]), accuracy (recovery), and uncertainty factor (Chandran and Singh 2007; Kristiansen 2003). The calibration curve was constructed in the presence of potato chips spiked samples with AA and AA‐d3 as internal standards to overcome the matrix effect.

The calibration curve was constructed by plotting the ratio of AA area to AA‐d3 area and AA concentration (ppb). The AA spike levels for constructing the calibration curve were 40–400 ppb (40, 50, 100, 250, and 400 ppb), and the AA‐d3 concentration was 250 ppb. Each concentration was prepared three times.

### 2.7 Quality Control Samples (QC)

Three samples of spiking potato chips at concentrations of 50, 250, and 400 ppb were used to assess the accuracy and precision variations within and between days of the suggested method.

### 2.8 Risk Assessment

#### 2.8.1 Noncarcinogenic Risk

To assess the noncarcinogenic risk from Chronic Daily Intake (CDI), two crucial factors influence the CDI: (1) AA concentration in food, (2) the amount of food per day and body weight. Therefore, in this study, seven categories of age and gender were evaluated. The CDI was calculated separately for each population group using Equation (1):

(1) $$\mathrm{CDI}=\frac{C\times \mathrm{IRi}\times \mathrm{EDi}\times \mathrm{EFi}}{\mathrm{BW}\times \mathrm{AT}}$$

The abbreviations in this equation are: CDI, the chronic daily intake (mg/kg/day); EFi, exposure frequency (365 days/year); EDi, exposure duration (Bacigalupo and Hale 2012); IRi, ingestion rate (19.33 g/person/day in Iran; Schripsema and Meijer 2017) as data for chips consumption is not available for each category in Iran, we assumed that all age groups consumed 19.33 g of potato chips per day. C is the AA concentration in chips (mg/kg), BW is the consumer body weight, which was determined based on data from the population within the seven age groups and sex, as reported by Azizi et al. (2002); and AT is the average exposure time for noncarcinogens (365 days/year * EDi).

The Target Hazard Quotient (THQ) is a noncancer risk assessment parameter that determines the potential health risks for hazard classes other than carcinogenicity of AA (Storelli 2008). The noncarcinogenic risk to consumers of the chips tested in the present study was estimated by Equation (2) (United States Environmental Protection Agency [USEPA] 2000; Yazdanpanah et al. 2022):

(2) $$\mathrm{THQ}=\frac{\mathrm{CDI}}{\mathrm{RfD}}$$

where THQ is the target hazard quotient; CDI is chronic daily intake, RfD or Reference Dose is the daily oral reference dose (mg/kg/day), for AA RfD is 0.002 (mg/kg/day), which does not differ between adults and children (IRIS 2012). The population in each category is at significant noncarcinogenic risk when THQ is higher than 1 and they need consideration (U.S. EPA 2011).

#### 2.8.2 The Margin of Exposure (MOE) Approach

European Food Safety Authority (EFSA) report describes the risk assessment of unavoidable genotoxic food carcinogens such as aflatoxin B1, AA, and PAH using the Margin of Exposure (MOE) approach (Barlow and Schlatter 2010; European Food Safety Authority (EFSA) 2005). The calculated chronic intake values were used to perform a risk characterization of AA through the assessment of MOE as follows (Esposito et al. 2017; European Food Safety Authority (EFSA) 2005):

(3) $$\mathrm{MOE}={\text{BMDL}}_{10}/\mathrm{DI}$$

MOE = Margin of Exposure (dimensionless); BMDL_10_ = Benchmark Dose Lower Confidence Limit for a 10% response (mg/kg BW/day); DI = Daily Intake of AA previously calculated (mg/kg/day).

#### 2.8.3 Estimation of Carcinogenic Risk

The carcinogenic risk for each category of consumers due to consumption of chips in different years was estimated by using Incremental Lifetime Cancer Risk (ILCR) and calculated by Equation (4) (Shahrbabki et al. 2018; U.S. EPA 2005):

(4) ILCR=CDI×CSF

In this equation, CDI is the chronic daily intake (mg/kg/day); CSF is the Cancer Slope Factor (mg/kg/day) as the risk caused by a lifetime average dose of 1 mg/kg BW/day. CSF for AA is 0.5 (mg/kg/day; IRIS 2012).

#### 2.8.4 New Approach to Risk Assessment

An alternative approach named Source Related HQs (HQs) was calculated, where HQs is the exposure ratio from the specific source of interest to their respective reference levels. According to this approach, the HQs should be adjusted by a correction factor before comparison with the reference dose to simulate aggregate exposure. A correction factor was calculated based on the permitted exposure contribution from the specific source to the permitted aggregated exposure (Goumenou and Tsatsakis 2019). An extrapolation from the specific source exposure to the aggregated was suggested using the legally permitted exposures to get around the challenge of estimating actual aggregated dietary exposure. Aggregate exposure is the sum of permitted exposure from all related food items (Goumenou and Tsatsakis 2019)

(5) $$\mathrm{EXP}\;\text{aggregated}=\frac{\text{Exposure from specific food items}}{\mathrm{CF}}$$

(6) $$\mathrm{CF}\;\left(\text{correction factor}\right)=\frac{\text{Permitted exposure from the specific food item}\;}{\mathrm{Sum}\;\text{of permitted exposure from}\;\mathrm{all}\;\text{related food items}}$$

In the exposure assessment, the average consumption rate of potato chips, soft bread, breakfast cereal, sweets, instant coffee, and roast coffees in Iran is estimated at 19.33, 320,130, 8, 0.38, and 0.38 g per person per day for adults, respectively (FAOSTAT 2024; National Nutrition and Food Technology Research Institute 2004; Nikolic et al. 2011; Schripsema and Meijer 2017). Since there is no available data for the consumption of potato chips, soft bread, breakfast cereal, and sweets by children in Iran, consumption of potato chips, soft bread, breakfast cereal, and sweets was assumed to be 19.33, 160, 65, and 8 g per person per day, respectively (Schripsema and Meijer 2017; Yazdanpanah et al. 2022). The daily consumption of processed cereal‐based baby foods per Iranian is 100 g (Mirza Alizadeh et al. 2023). The benchmark level of 750 ppb for potato chips, 100 ppb for soft bread, 300 ppb for breakfast cereal, 350 ppb for biscuits, 400 ppb for crackers, crisp bread, and similar, 850 ppb for instant coffee, 400 ppb for roast coffees and 150 ppb for processed cereal‐based baby foods were used (European Commission 2017).

(7) $$\frac{\left(\mathrm{EXP}\;\text{from specific food item}\right)/\mathrm{CF}}{\mathrm{ADI}}<1$$

ADI is the acceptable daily intake (mg/kg/day) in this equation.

Which is equal to:

(8) $$\frac{\mathrm{HQs}}{\mathrm{CF}}<1$$

HQs is source‐related hazard quotient in this equation. This equation means, eventually that for no risk we should have:

(9) HQs<CF

### 2.9 Statistical Analysis

The analyzed data were displayed as mean ± SD (standard deviation), median, 90th percentile, and 97.5th percentile. In this research, the risk assessment investigations were performed using Excel 2021.

## 3 Result

### 3.1 Method Validation

The calibration curve was linear over a concentration range of 40–400 ppb (40, 50, 100, 250, and 400 ppb) and an AA‐d3 concentration of 250 ppb with a high correlation coefficient (*r* ^2^ = 0.9999).

Interday and intraday accuracy and precision were evaluated using three spiked potato chip samples at concentrations of 50.0, 250.0, and 400.0 ppb on 3 days. The accuracy ranged from 98.41% to 100.17%, and the coefficient of variation (%CV) was less than 9.71% (Table 1).

**TABLE 1:** Performance parameters of the optimized method: Recovery, coefficient of variation (CV), and uncertainty at different spike levels.

Spike level (ng/g)	Average of recovery (%)	CV (%)	Uncertainty (%)
50	98.4	9.7	19.4
250	100.2	9.1	18.1
400	98.2	8.1	16.1

The accuracy and the precision were both within the standard range of European guidelines (European Commission 2019). The expanded measurement uncertainty was calculated with a coverage factor of 2, resulting in a confidence level of about 95% (U = 2u) (Table 1; Commission 2015).

The limit of detection (LOD) and the limit of quantification (LOQ) were determined to be 5.0 and 40.0 ng/g, which fall within the acceptable range of European Commission regulation. According to the European Commission regulation, the acceptable limit of quantification (LOQ) for food items with AA benchmark levels exceeding 125 μg/kg is less than or equal to 50 μg/kg, and the LOD level is one‐third of the LOQ (European Commission 2019).

### 3.2 Determination of AA in Potato Chips Samples

The occurrence of AA in potato chip samples is summarized in Table 2. Among the samples analyzed, 13% of those collected in 2016–2017 and 31% of those collected in 2020–2021 were found to contain detectable levels of AA. The average AA concentrations were 25.01 ppb in 2016–2017 and 31.09 ppb in 2020–2021.

**TABLE 2:** Occurrence of AA in potato chips.^a^

Date of sampling (Number of sample)	Mean ± SD (ng/g)	Median	90th percentile	97.5th percentile	Number of samples in the range (ng/g)	
					< 40	> 40
2016–2017 (*N* = 69)	25.1 ± 13.4	20	45.9	56.9	60	9
2020–2021 (*N* = 44)	31.9 ± 18.9	26.8	64.5	73.5	30	14

^a^ Data below LOQ (40 ng/g) has been assumed to be 20 ng/g.

According to the Commission Regulation (EU) 2017/2158, the benchmark level for AA in potato chips is 750 μg/kg (ppb). All 113 tested potato chip samples in this study had AA concentrations well below this benchmark level (European Commission 2017).

### 3.3 Dietary Exposure

Chronic dietary exposure is estimated for different genders and age groups (from children aged 3–4 years to adults aged 64 years and older) in different years. Consumption for all age and gender groups is estimated at 19.33 g/person/day (Schripsema and Meijer 2017).

**FIGURE 1:**
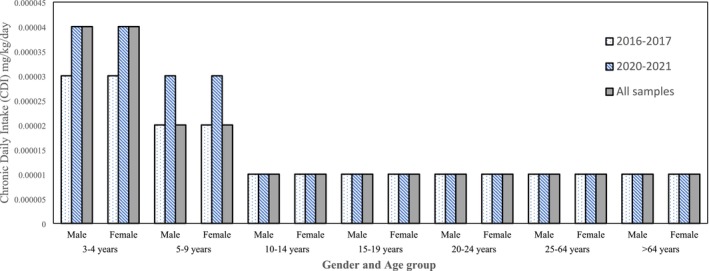
Estimated dietary exposure to AA through potato chips consumption in the Iranian population (data below LOQ (40 ng/g) have been assumed to be 20 ng/g).

The CDI for males and females was the same in all categories, but AA intake from chips in children was higher than in adults. Children between the ages of three and four have a CDI four times higher than that of adults. For children aged 5–9 years, CDI was twice as high as for adults. The CDI for children in 2020–2021 is higher than that for 2016–2017, as shown in Figure 1. During 2016–2017, the CDI was 0.00003 for the first category and 0.00002 for the second category (5–9 years). The AA intake in other categories was 0.00001. In 2020–2021, children aged 3–4 had an AA intake three times higher than that of adults, and children aged 5–9 had an AA intake of 0.00002, twice that of adults. This calculation shows that higher AA intake was due to lower body weight in children.

### 3.4 Risk Assessment

#### 3.4.1 Noncarcinogenic Risk of AA

The THQ parameter was used to calculate the AA risk for the Iranian population based on their consumption of potato chips during two distinct study years. The results are displayed in Table 3 for males and females in separate age groups.

**TABLE 3:** Risk assessment parameters for acrylamide (AA) exposure from potato chip consumption in the Iranian population: Target Hazard Quotient (THQ), Incremental Lifetime Cancer Risk (ILCR), and Margin of Exposure (MOE).

Date of sampling	Gender and Age group (Year)	3–4		5–9		10–14		15–19		20–24		25–64		> 64	
		Male	Female	Male	Female	Male	Female	Male	Female	Male	Female	Male	Female	Male	Female
2016–2017	THQ	0.01604	0.01612	0.01060	0.01053	0.00581	0.00575	0.00383	0.00436	0.00342	0.00410	0.00322	0.00351	0.00343	0.00376
	ILCR	0.00002	0.00002	0.00001	0.00001	0.00001	0.00001	0.00000	0.00000	0.00000	0.00000	0.00000	0.00000	0.00000	0.00000
	MOE (0.17)	5298.51	5273.97	8016.15	8072.25	14,629.65	14,783.94	22,196.95	19,475.80	24,854.97	20,720.66	26,397.89	24,220.27	24,795.36	22,600.21
	MOE (0.43)	13,402.12	13,340.03	20,276.14	20,418.06	37,004.40	37,394.67	56,145.22	49,262.33	62,868.45	52,411.07	66,771.12	61,263.04	62,717.67	57,165.23
2020–2021	THQ	0.02037	0.02047	0.01347	0.01337	0.00738	0.00730	0.00486	0.00554	0.00434	0.00521	0.00409	0.00446	0.00435	0.00478
	ILCR	0.00002	0.00002	0.00001	0.00001	0.00001	0.00001	0.00000	0.00001	0.00000	0.00001	0.00000	0.00000	0.00000	0.00000
	MOE (0.17)	4172.27	4152.94	6312.25	6356.43	11,519.99	11,641.48	17,478.79	15,336.05	19,571.83	16,316.30	20,786.78	19,072.04	19,524.88	17,796.33
	MOE (0.43)	10,553.38	10,504.49	15,966.27	16,078.02	29,138.79	29,446.10	44,211.05	38,791.18	49,505.21	41,270.64	52,578.33	48,241.04	49,386.47	45,014.26
All samples	THQ	0.01772	0.01781	0.01172	0.01163	0.00642	0.00635	0.00423	0.00482	0.00378	0.00453	0.00356	0.00388	0.00379	0.00416
	ILCR	0.00002	0.00002	0.00001	0.00001	0.00001	0.00001	0.00000	0.00000	0.00000	0.00000	0.00000	0.00000	0.00000	0.00000
	MOE (0.17)	4795.62	4773.41	7255.32	7306.10	13,241.12	13,380.77	20,090.20	17,627.33	22,495.95	18,754.03	23,892.42	21,921.49	22,441.99	20,455.19
	MOE (0.43)	12,130.10	12,073.91	18,351.70	18,480.15	33,492.25	33,845.48	50,816.39	44,586.76	56,901.51	47,436.66	60,433.77	55,448.47	56,765.04	51,739.59

In 2020–2021, the THQ parameter was approximately 20% higher than in 2016–2017. Table 3 illustrates that the risk associated with AA exposure decreases with age, indicating that younger individuals are more vulnerable. Additionally, the risk of noncarcinogenic effects from AA may vary between the sexes. Based on the analysis of all sample results, the THQ value indicates potential noncarcinogenic risk, with all values below 1, suggesting that no age group faces significant noncarcinogenic concerns. However, children aged 3–4 years and 5–9 years consistently have the highest THQ values, making them the most vulnerable groups for noncarcinogenic effects of AA exposure. Adolescents aged 10–14 and 15–19 years show lower THQ values than the younger age groups but remain higher than adults, indicating a moderate level of risk. In contrast, adults across all age ranges (20–24, 25–64, and over 64) exhibit the lowest THQ values, suggesting they are at the least risk for noncarcinogenic effects.

#### 3.4.2 MOE of Acrylamide

The risk assessment of AA for genotoxic and carcinogenic effects was considered using the MOE ratio. In this ratio, the BMDL_10_ value is assumed to be 0.43 mg/kg body weight per day for peripheral nerve toxicity based on information from animal studies and its translation into human studies; the lowest BMDL_10_ of 0.17 mg/kg body weight per day is considered for genotoxic effects, which are computed for two distinct years in Table 3.

According to the EFSA's report, given the general uncertainties in interpreting substances that are both genotoxic and carcinogenic from a public health perspective of little importance, a MOE would be 10,000 or more based on a BMDL10 from an animal study (Commission 2015).

Based on the data calculated for the MOE for males and females under 10 years old in both sampling years, the neoplastic MOE (0.17 mg/kg) was < 10,000 (Table 3), Thus, the results indicate that the neoplastic effects of AA through potato chips consumption can be threatening in both genders under 10 years old as the MOE is below 10,000 and could fall into the category of highest public health concern.

#### 3.4.3 Carcinogenic Risk Estimation of Acrylamide

USEPA announced 1.00 E‐5 as an acceptable ILCR (the risk of developing cancer during the lifetime in the exposed population is 1 in 100,000; IRIS 2012).

Table 4 shows the estimated incremental lifetime cancer risk (ILCR) in the Iranian population by age and year based on AA consumption of potato chips. The calculated ILCR shows a decrease with increasing age in 2016–2017; children aged 3–4 years old have a score of 2.00E‐5, and children in two other categories (5–9 and 10–14 years) have a maximum acceptable ILCR (1.00 E‐5). In 2020–2021, children aged 3–4 years have the same ILCR as in 2016–2017, which is twice the USEPA's acceptable limit, and in other child categories (5–9, 10–14 years), they also have the same value as 2016–2017. When evaluating all samples, there was a decline in the order: children (3–4) > children (5–9) = children (10–14) > over 15 years. The results show that children were at higher risk of cancer during their lives.

#### 3.4.4 New Approach to Risk Assessment

Consumption of the food, maximum permitted exposure to AA for the main food items is calculated based on the benchmark level, HQ and HQs in adults and children are shown in Tables 4 and 5.

**TABLE 4:** Consumption of the food, maximum permitted exposure to acrylamide for the main food items, HQ, and HQs in adults.

Food items	Consumption of food (g/kg bw/day)	Maximum permitted exposure (ng/kg bw/day)	HQ	HQs
Potato chips samples (2016–2017)	0.28	207.11	0.0035	0.0212
Potato chips samples (2020–2021)	0.28	207.11	0.0044	0.0270
All Potato chips samples	0.28	207.11	0.0038	0.0235
Soft bread	4.58	457.14		
Breakfast cereal	1.86	557.14		
Biscuits, crackers, crisp bread, and similar	0.11	45.71		
Instant coffee	0.0055	4.66		
Roast coffees	0.0055	2.19		
The sum of maximum permitted exposure (ng/kg bw/day)		1273.96		

**TABLE 5:** Consumption of the food, maximum permitted exposure to Acrylamide for the main food items, HQ and HQs in children.

Food items	Consumption of food (g/kg bw/day)	Maximum permitted exposure (ng/kg bw/day)	HQ	HQs
Potato chips samples (2016–2017)	1.29	966.50	0.0161	0.0758
Potato chips samples (2020–2021)	1.29	966.50	0.0205	0.0966
All Potato chips samples	1.29	966.50	0.0179	0.0840
Soft bread	10.67	1066.67		
Breakfast cereal	4.33	1300.00		
Biscuits, crackers, crisp bread, and similar	0.53	213.33		
Processed cereal‐based baby foods	6.67	1000.00		
The sum of maximum permitted exposure (ng/kg bw/day)		4546.5		

The calculated source‐specific correction factor (CF) was 0.16 and 0.21 for the adults and children, respectively, according to Equation (6). Tables 4 and 5 demonstrate that the calculated values for the HQs of 0.00384 and 0.0179 (for adults and children, respectively) were significantly lower than the corresponding values for the HQs of 0.0235 and 0.0840, indicating that there was no risk of AA exposure for the Iranian population who consume potato chips.

## 4 Discussion

### 4.1 Method Validation

Low levels of AA in a full‐fat matrix, such as potato chips, are challenging to analyze. Following the theory of similarity miscibility, AA is extracted using polar media. These polar media include water, solutions such as formic acid, and organic solvents like methanol, acetonitrile, and acetone (Commission 2015; Hu et al. 2015). Methanol was used as the extraction solvent in this method, as it helps avoid the extraction of starch and certain polysaccharides. It has also been effective in precipitating and eliminating proteins, resulting in a clearer extract than water, even without the use of centrifugation. Furthermore, methanol can be readily vaporized with a gentle stream of nitrogen to enhance the limit of quantification (LOQ) through concentration (Gökmen and Şenyuva 2006). The extraction volume in QuEChERS procedures for the determination of AA in potato chips is 20, 8, and 10 mL, respectively, in the literature by Bertuzzi et al. (2017), Cheng et al. (2019) and Stefanović et al. (2017) this is much more than 2.5 mL, which was used in this investigation. To precipitate and purify protein‐rich food samples, precipitation, and protein removal were carried out using Carrez reagents ([I] hexacyanoferrate (II) and [II] zinc sulfate), acetone, ethanol, or methanol (Gökmen and Şenyuva 2006; Hu et al. 2015).

Sample purification using SPE cartridges is a routine procedure to enhance the selectivity of standard methods in LC–MS analysis after extraction. However, this step may increase the overall cost and complexity of operations (Hu et al. 2015; Yazdanpanah et al. 2022). The validated approach of substituting PSA for the sorption bed and introducing the sample solution was demonstrated in the published article (Eslamizad, Kobarfard, Tabib, et al. 2019). Using PSA as a sorbent bed is one of the advantages of this method because DSPE‐based techniques allow the reduction of extra steps, such as centrifugation, filtration, and precipitation, which minimizes sample manipulation and multistage preparation.

An additional advantage of utilizing this approach involves incorporating an internal standard and constructing a calibration curve with AA and AA‐d3 spiked potato chip samples. The inclusion of an internal standard in food samples, along with a spiked calibration curve, helps to avoid potential AA losses during sample preparation and overcomes the effect of the matrix (Commission 2015; Hooshfar et al. 2020). Consequently, an enhancement in the accuracy, precision, and repeatability of the measurements was noted with the implementation of this technique.

Determining AA in food using GC–MS methods mostly require derivatization, which is time‐consuming and complex, and also GC–MS shows many false positives while measuring AA due to the high temperature of the injectors (Zhang et al. 2005). These factors led to selecting the LC–MS technique for AA analysis in this investigation.

### 4.2 Incidence of Acrylamide in Potato Chips

The results showed that the mean AA concentration in this study was 25.1 ppb in 2016–2017 and 31.9 ppb in 2020–2021, indicating a slight attenuation over several years.

As shown in Table 6, the mean concentration of AA in this study for all samples was about four times higher than the values in the Lim H.H study (Lim and Shin 2014), within the range of two studies (Chen et al. 2012; Mesias et al. 2018), and lower than the remaining studies. The mean value of AA in this study was about 14 times (13.77) lower than the EFSAs report in 2015 from 31,467 potato chip samples from different studies (European Food Safety Authority (EFSA) 2015).

**TABLE 6:** Comparative techniques for analyzing acrylamide (AA) in samples of potato chips from different studies.

Row	Country	Matrix	LOD (ng/g)	LOQ (ng/g)	Number of Samples	AA range (ng/g)	Mean AA (ng/g)	Analytical instrument	References
1	Iran	Potato chips	5	40	113	20–90.01	28.46	LC–MS/MS	This study
2	Korea	Potato chips	0.04	0.14	12	0.4–14.3	5.5	LC–MS/MS	Lim and Shin (2014)
3	Iran	Potato chips	0.6	2	4	68–89	—	LC–MS/MS	Zokaei et al. (2016)
4	China	Potato chips	2.4	—	11	17.39–398.23	137.91	LC–MS/MS	Chen et al. (2012)
5	Chile	Potato chips	2.69	8.99	3	—	354.13	GC‐MC	Barrios‐Rodríguez et al. (2021)
6	Spain	Potato chips	4	12	6	105–860	—	LC–MS/MS	Ferrer‐Aguirre et al. (2016)
7	Ethiopia	Potato chips	5	20.40	30	211–3515	1298	HPLC‐DAD	Deribew and Woldegiorgis (2021)
8	Iran	Potato chips and corn products	< 10	< 30	12	244–1688	—	LC–MS/MS	Boroushaki et al. (2010)
9	Spain	French fries	—	20	73	24–3641	644	LC–MS	Mesias et al. (2018)
10	UK	Potato chips	—	—	20	131–5360	—	LC–MS/MS	Elmore et al. (2015)
11	Spain	Potato chips	—	—	80	108–2180	630	—	Mesías and Morales (2015)
12	EU	Potato chips	—	—	280	574–576	—	LC–MS/MS	EFSA (2011)
	EU				458	626–630		LC–MS	
	EU				388	689–693		GC–MS	
13	EU	Potato chips	—	—	7	220–1456.67	—	LC–MS–MS	Saeidi Asl et al. (2014)
14	EU	Potato chips	—	—	—	435–8825	—	GC–MS	Shojaee‐Aliabadi et al. (2013)

### 4.3 Dietary Exposure

EFSA has reported an average chronic dietary exposure to AA for various food product categories based on age groups; the most exposed groups were toddlers and children, with 0.5–1.9 ppb body weight per day, while 0.4 to 0.9 ppb body weight for adults, adolescents, and the elderly each day. Grain‐based foods, other potato‐based products, soft breads, breakfast cereals, cookies, crackers, crispbreads, and coffee accounted for the largest share of total exposure to AA (European Food Safety Authority (EFSA) 2015).

In the Eslamizad, Kobarfard, Tabib, et al. (2019) study on the determination of AA content in different types of bread, daily dietary exposure of AA for all samples was 127 (ng/kg bw/day). In the study conducted by Nematollahi and colleagues, dietary exposure to AA was assessed for seven types of food containing AA (bread, baked goods, confectionery, snack food, fast food, roasted nuts, and coffee) among different age groups of the Tehran population. Their studies showed 1.81 μg/kg body weight/day for children aged 3–10 years, 1.02 μg/kg body weight/day for adolescents aged 11–17 years, 0.61 μg/kg body weight/day for adults aged 18–60 years, and 0.53 μg/kg body weight/day for seniors aged 61–96 years. For all the age groups in this study, the highest and lowest intake rates (g/day) were observed for bread and coffee, respectively, since bread contributed the highest proportion due to its high consumption rate (Nematollahi et al. 2020). In a study conducted in Chile in 2021, the 43 most commonly consumed thermally treated starchy foods were selected and divided into six groups: bread, potato chips, breakfast cereal, Coca‐Cola, biscuits, and others. The EDI (Estimated daily intake) of AA in the metropolitan region of Santiago was reported to be 0.98 μg/kg body weight/day (Barrios‐Rodríguez et al. 2021).

The study highlights the same concerns, as the aforementioned studies show that children are at higher risk from chronic AA dietary intake from potato chip consumption than adults. Controlling AA content in food products is a big challenge for food manufacturers and consumers. Therefore, using various strategies to reduce the formation of this compound, such as choosing the suitable variety of potato, storing at temperatures above 8°C, lactic fermentation, frying under vacuum, or at temperatures lower than 120°C, or soaking potato chips in acetic acid, citric acid, glycine, hydrocolloids, salts, asparaginase enzyme, vitamins, and antioxidants can help to obtain safer potato chips (Abedini et al. 2024; Mousavi et al. 2024).

### 4.4 Risk Assessment

Based on the results, children of both sexes aged between 5 and 9 years and between 3 and 4 years in Iran who consume potato chips have the highest potential health risk from AA intake. The HQ and HQs for the potato chips dietary intake were below one. HQs was about six times higher than HQ in adults and about four times higher in children because other foods contribute to the dietary intake of AA.

Several studies regarding the risk assessment of AA from a single food item indicate a low health risk (Aghvami et al. 2023; Faraji et al. 2024; Seilani et al. 2021), but numerous examples demonstrate that exposure to a variety of sources over time may give rise to unanticipated harmful mixture effects, even at low concentrations below recognized safe thresholds (Mititelu et al. 2022). Although all values are less than 1, which do not represent a risk generally, humans are exposed to thousands of different chemicals originating from different sources, which may exert additive or even synergistic effects (Tsatsakis et al. 2017).

Also, the results of the present study indicate that the Incremental Lifetime Cancer Risk (ILCR) of AA among children followed in descending order: 3–4 years old > 5–9 years old > 10–14 years old due to consumption of potato chips is higher than the value 1.00E‐5. The MOE results show that eating potato chips can have neoplastic effects on both sexes, but it can be hazardous for children under 10 years old because the MOE is less than 10,000 and may be considered a high public health concern.

In Nematollahi et al.'s study, related potential risks for AA were estimated. The THQ was less than 1 for 7 groups of heated foods, representing a negligible risk among all age categories, and the estimated ILCR greater than 10^−4^ for all age categories indicated a serious risk to the population. Also, the MOE values based on carcinogenicity presented health concerns in all age groups (< 10,000). In their study, bread, despite having a low AA content (157 ppb compared to other nuts, except for roasted nuts), due to its high consumption rate, has the highest proportion in all age groups (Nematollahi et al. 2020).

Hoseini Majd et al. (2025) investigation on human health risk assessment of AA through consuming Iranian Biscuits indicated that a serious noncarcinogenic risk (95th percentile; THQ > 1) for children and a carcinogenic risk (ILCR > 1E‐4) for children and adults.

Esposito et al. (2017) found that MOE was less than 10,000 for every age group, except the elderly and very elderly. Furthermore, the research by Nematollahi et al. (2020) indicates that all age groups (less than 10,000) had health concerns based on the MOE values based on carcinogenicity. In terms of comparison, in our study, the MOE for non‐neoplastic effects for all age groups showed little concern as they were above 10,000, but for neoplastic effects, children under 10 were highly concerning for public health.

## 5 Conclusion

This study evaluated the risk of AA exposure in the Iranian population, including males and females for various age groups who consumed potato chips in two distinct years, 2016–2017 and 2020–2021. The results showed that the chronic cumulative risk assessed by THQ and HQs had no concern for AA levels (THQ or HQs < 1). The estimated ILCR from AA for children aged 3–4, 5–9, and 10–14 years has been reported to be more than 1.00 E‐5, indicating an increased risk of cancer in the population. In addition, the results suggest that the non‐neoplastic effects of AA in potato chips may be threatening in men and women under the age of 10, as the MOE is below 10,000 and could fall into the highest public health concern category.

The presence of AA in trace amounts may have negative health effects when combined with other substances for an extended period, given the current state of being exposed to a variety of chemical combinations from various sources, such as the environment, diet, consumer goods, and water consumption. Thus, it is suggested to conduct additional research to track the presence of AA in different food products, evaluate the average dietary intake, and identify any health risks associated with AA in Iran's main food supplies. These results provide useful information for regulatory authorities, food manufacturers, and consumers to make informed decisions about food safety and dietary habits.

## Ethics Statement

The authors have nothing to report.

## Conflicts of Interest

The authors declare no conflicts of interest.

## Data Availability

The data that support the findings of this study are available on request from the corresponding author. The data are not publicly available due to privacy or ethical restrictions.
